# Alamandine reduces leptin expression through the c-Src/p38 MAP kinase pathway in adipose tissue

**DOI:** 10.1371/journal.pone.0178769

**Published:** 2017-06-07

**Authors:** Tsuyoshi Uchiyama, Fumikazu Okajima, Chihiro Mogi, Ayaka Tobo, Shoichi Tomono, Koichi Sato

**Affiliations:** 1 Laboratory of Signal Transduction, Institute for Molecular and Cellular Regulation, Gunma University, Maebashi, Gunma, Japan; 2 Aomori University, Faculty of Pharmaceutical Sciences, Aomori, Japan; 3 Gunma University School of Health Sciences, Maebashi, Gunma, Japan; Max Delbruck Centrum fur Molekulare Medizin Berlin Buch, GERMANY

## Abstract

**Objective:**

Obesity is associated with an increased risk of diabetes mellitus, hypertension, and renal dysfunction. Angiotensin 1–7 and alamandine are heptameric renin angiotensin system peptide hormones. Further, alamandine levels increase with renal dysfunction. In the cardiovascular system, angiotensin 1–7 and alamandine produce similar improvements and counterbalance angiotensin II in regulating vascular function. We aimed to determine whether the effect of alamandine on leptin expression and secretion in adipocytes was similar to that of angiotensin 1–7.

**Approach and results:**

We studied isolated peri-renal visceral adipose tissue and peri-renal isolated visceral adipocytes from male Wistar rats. Angiotensin II from 0.01 to 10nM had no effect on leptin expression. Angiotensin 1–7 (1 nM) increased leptin secretion and expression, whereas alamandine (1 nM) decreased leptin secretion and expression in adipose tissue and isolated adipocytes and reduced blood leptin levels *in vivo*. These effects were mediated by Gq, c-Src, p38 mitogen-activated protein, and IκB activation. Additionally, alamandine induced nitric oxide expression via inducible nitric oxidase synthase and plasminogen activator inhibitor 1 expression in adipose tissue and isolated adipocytes.

**Conclusions:**

Angiotensin 1–7 and alamandine produced opposing effects on leptin expression and secretion in adipose tissue. This result suggests that the action of Mas (angiotensin 1–7 receptor) and Mas-related G-protein coupled receptor D in adipocytes exhibited opposing actions similar to angiotensin II type 1 and type 2 receptors.

## Introduction

The renin angiotensin system (RAS) regulates circulating blood volume and blood pressure, and is involved in vascular endothelial function and cardiac muscle hyperplasia [1–3]. Angiotensin II (AngII) is an octameric peptide hormone that is a RAS component. There are two kinds of AngII receptor, which have opposing actions [3].

Additionally, angiotensin 1–7 (Ang1-7) and alamandine are heptameric RAS peptide hormones [4, 5] (S1A Fig) that differ in a single C-terminal amino acid. The Ang1-7 receptor was identified as the Mas receptor in 2003 [6, 7], and the alamandine receptor was identified as the Mas-related G protein-coupled receptor D (MrgD) in 2013 [5, 8].

MrgD receptors are mainly expressed in dorsal root and sensory ganglia; moderately expressed in testes, urinary bladder, artery, and uterus; and slightly expressed in cerebellum, brown adipose, white adipose, and gastrointestinal tract [9] [10] [11]. Meanwhile, Mas (Ang1-7 receptor) is more broadly expressed than most other Mrg receptors.

MrgD and Mas elicit the same effects, namely improved cardiovascular function, which has a vascular component and an anti-fibrillation effect on cardiac muscle [12–16]. Leptin is mainly secreted by adipocytes and reflects the degree of adipocyte differentiation. Insulin, glucose, and glucocorticoids promote leptin release from adipocytes [17, 18]. Conversely, androgenic hormone and the sympathetic nerve system suppress leptin release [19]. Leptin controls the appetite center, and its release from adipocytes is diminished following β-adrenergic receptor activation by sympathetic nerves. This feedback system regulates serum leptin levels [20]. Leptin acts on tissues via two pathways; namely, direct effects mediated by activation of the leptin receptor, and indirect effects through sympathetic nerve activation [21]. In hypertension, leptin induces endothelial nitric oxide synthase expression and vasodilation by the direct action of leptin on vascular endothelial cells [22]. However, the activation of sympathetic nerves increases blood pressure, and the net effect of leptin is the induction of arteriosclerosis [23, 24].

Metabolic syndrome is characterized by visceral adiposity, insulin resistance, dyslipidemia, hypertension, and diabetes mellitus [25] [26]. Thus, we employed visceral adipose tissue to investigate whether the effects of MrgD receptor stimulation were similar to Mas receptor stimulation upon leptin expression in visceral adipose tissue.

There are reports investigating the influence of Ang1-7 on adipose tissue [27], [28]. In these reports, long-term Ang1-7 administration was observed to reduce the volume of adipose tissue [28]. Serum leptin levels were decreased in transgenic rats showing high blood concentrations of Ang1-7 [27]. However, Ang1-7 has a vasodilatation effect, and the indirect action on adipose tissue by Ang1-7 may contribute to the improvement of metabolism and the reduction of adipose tissue volume and serum leptin levels with long-term dosage. In contrast, we investigated the direct effects of Ang1-7 and alamandine on leptin expression and secretion in adipose tissue and isolated adipocytes.

The RAS has a complicated synthetic pathway, with two possible pathways involved in generating alamandine (S1B Fig). Alamandine can be synthesized from either Ang1-7 or angiotensin A [15]. Leukocyte-derived aspartate decarboxylase can replace the asparagine of the amino terminal of Ang1-7 with alanine [29], whereas angiotensin converting enzyme 2 can cleave the C-terminal phenylalanine of angiotensin A [5]. AngII receptor type 1 blockers and angiotensin converting enzyme inhibitors increase angiotensin converting enzyme 2 expression [30, 31] and are commonly prescribed as antihypertensive drugs for diabetic and hypertensive patients. Clinically, the blood levels of both angiotensin A and alamandine increase with renal dysfunction [5, 32]. Because alamandine can be synthesized from Ang1-7, serum levels of alamandine are regarded as being equivalent to Ang1-7, which are normally ≈ 20 pM [33]. We performed most of our experiments with 1 nM alamandine to closely approximate *in vivo* conditions.

## Materials and methods

### Ethics statement

This study was carried out in strict accordance with the guidelines of the Animal Care and Experimentation Committee of Gunma University. All experimental procedures were performed in accordance with the guidelines of the animal care and experimentation committee of Gunma University. The protocol was approved by the Animal Care and Experimentation Committee of Gunma University (Permit Number: 14–29). Rats were sacrificed using diethyl ether and all efforts were made to minimize suffering.

### Materials

DMEM, FBS, calf serum, collagenase type I, and Opti-MEM were purchased from Invitrogen (Grand Island, NY). Insulin was purchased from the Cell Science and Technology Institute, Inc., (Miyagi, Japan). Alamandine and D-Pro^7^ angiotensin1-7 were purchased from Phenix Pharmaceuticals, Inc. (Burlingame, CA). Mouse 3T3L-1 cells were kindly provided by Dr. Hiroshi Shibata of Gunma University (Gunma, Japan). SB239063 was purchased from R&D Systems, Inc., (Minneapolis, MN). N^G^-nitro-arginine methyl ester hydrochloride (L-NAME), 1400w, AG490, BAY11-7082, candesartan, S-nitroso-L-glutathione (GSNO), and PP2 were purchased from Cayman Chemical (Ann Arbor, MI).

Antibodies against c-Src, p38 MAP kinase, and IκB*α* (32, 36) were purchased from Cell Signaling (Beverly, MA). Antibody against β-actin was purchased from Gene Tex, Inc., (Irvine, CA). Antibody against plasminogen activator inhibitor 1 was purchased from Cusabio Biotech Co., Ltd., (College Park, MD). Angiotensin 1–7, TRI Reagent, protease inhibitor, phosphatase inhibitors I and II, A779, PD123177, and isobutyl methyl xanthine were purchased from Sigma Aldrich (St. Louis, MO). YM25490 was a gift from Dr. H Taniguchi of Astellas (Tsukuba, Japan). Pertussis toxin (PTX) was purchased from List Biological Laboratories Inc., (Campbell, CA). ECL Prime (GE Healthcare; Pittsburgh, PA) was used as the chemiluminescence detection system. Brilliant QPCR master mix and Brilliant SYBR Green master mix were purchased from Agilent Technologies (Santa Clara, CA). RT-PCR probes specific for rat were purchased from Applied Biosystems (Foster City, CA).

The sources of all other reagents were the same as described previously [34]. In the present study, we used many inhibitors and activators of intracellular signaling pathways. For convenience, their sites of action are summarized in S1 Table.

### Animals

Male Wistar rats, 10–13 weeks old, were purchased from Charles River Laboratory Japan (Yokohama, Japan). The rats were housed in a breeding room approved by the university with a 12 h light (07:00 to 19:00) / 12 h dark (19:00 to 07:00) light cycle. The rats were provided access to food and water *ad libitum*. The rats were maintained under the above conditions during the experimental period.

### Adipose tissue (AT) isolation and its culture

Rats were sacrificed using diethyl ether. AT was collected from the peri-renal visceral AT except for the upper part of the kidney. The collected AT was washed with Hanks’ balanced salt solution containing 1% BSA (fraction V), cut into pieces weighing approximately 100 mg, and cultured in 6-well plates containing 4.5 mL/well DMEM supplemented with 1% FBS and 1.72 μM insulin, at 37°C in a 5% CO_2_ incubator. The medium was changed the next day and the tissue was used for experiments 24 h thereafter.

### Isolation of adipocytes and their culture

AT was minced using a pair of scissors and vigorously shaken at 37°C in a water bath for 1 h with 1 mg/mL of collagenase type I. The tissue was then filtered using mesh with a 320 μm pore size. For preparation of adipocytes, the filtrate was washed three times with Hanks’ balanced salt solution containing 3% BSA (fraction V) without calcium and then finally washed with adipocyte culture medium composed of DMEM containing 1000 mg/dL glucose, 7% BSA (fraction V), 15 mM HEPES, 1.72 μM insulin, 5 μM adenosine, and 100 μM β-mercaptoethanol. The adipocytes in the upper layer were then cultured in 50 mL conical tubes with 20 mL culture medium per 5 mL of packed adipocytes, at 37°C in a 5% CO_2_ incubator overnight. The next day, 200 μL of packed adipocytes were placed into a 2 mL tube and cultured with 1 mL of adipocyte culture medium, at 37°C in a 5% CO_2_ incubator for 24 h.

### Animal treatments: *In vivo* examination procedure

Wistar rats (10-week-old) were bred as above, and the experiments were carried out 3 days after the purchase date to equalize body weights.

Bolus injection of alamandine, Ang1-7, or saline was administrated intraperitoneally twice at 576, 57.6, 5.76, or 0.576 μg/kg with 24 h between treatments [35] [36]. Twenty-four hours later, body weights were measured and the rats were anesthetized with diethyl ether. Blood samples were collected from tail vein. And both sides of the whole peri-renal AT were extracted, weighed, and then stored at -80°C until use. Blood samples were centrifuged at 2000 rpm and the serum was stored at -80°C until use.

### Measurement of leptin level

Leptin levels were determined using a leptin immunoassay kit (R&D Systems, Inc., Minneapolis, MN) according to the manufacturer’s protocol. The peri-renal AT was homogenized in lysate buffer (dilution buffer, included in the assay kit and protease inhibitor) at 500 μL per 100 mg of AT on ice and then centrifuged twice to remove lipids and debris. Rat serum was diluted 5 fold in dilution buffer, while AT was diluted 15-fold, and the supernatant containing adipocytes 4-fold.

### Plasmids expressing human Mas and MrgD receptors

Plasmids for the human Mas (GenBank accession no. M13150.1) and human MrgD (GenBank accession no. AY427820.1) receptors were obtained from Kazusa DNA Research Institute (Chiba, Japan).

The expression vectors were subcloned from their respective cDNAs by PCR using the following primers: for the human Mas receptor, 5′-CACCATGGATGGGTCAAACGTGACATCATTT-3′ (forward) and 5′-AAAGACGACAGTCTCAACTGTGACCGTATT-3′ (reverse; lacking a stop codon); for the human MrgD receptor, 5′-CACCATGAACCAGACTTTGAATAGCAGTGGG-3′ (forward) and 5′-AAAAGCCCCCATCTCATTGGTGCCCACGGT-3′ (reverse; lacking a stop codon).

The PCR products were cloned into the pcDNA3.1D/V5HisTOPO expression vector (Invitrogen) and subsequently sequenced. Protein expression was confirmed by western blotting in transfected differentiated 3T3L-1 adipocytes.

### Transfection to 3T3L-1 cells and differentiation to adipocytes

The transfection of plasmid into 3T3L-1 adipocytes was performed using the lipofection (Lipofectamine 2000; Invitrogen) method immediately prior to the addition of the differentiation agent. 3T3L-1 cells were plated at 2.0 x10^5^ cells/well in a 24-well plate in 1 mL of 4500 mg/L glucose DMEM supplemented with 10% calf serum, and incubated at 37°C in a 5% CO_2_ incubator for 12 h. Plasmid (0.5 μg) was mixed with 1 μL of Lipofectamine 2000 in 50 μL of Opti-MEM and added to each well. The plasmid solution was incubated for 5 min prior to addition to 3T3L-1 cells in 150 μL of serum-free DMEM and incubated for 2 h with gentle mixing every 15 min. Next, the initial differentiation agent (10% FBS, 1.72 μM insulin, 0.5 mM isobutyl methyl xanthine, 1 μM dexamethasone) in 1000 mg/L glucose DMEM was added and the samples were incubated at 37°C in a 5% CO_2_ incubator without changing the culture medium for 2 d. After the initial differentiation, the medium (10% FBS and 1.72 μM insulin in 1000 mg/L glucose DMEM) was changed every other day for about 8 d until morphological changes were observed (droplet accumulation in the adipocyte cells).

### Protein extraction and western blot analysis

After washing AT (approximately 100 mg), adipocytes (200 μL volume), or 3T3L-1 cells with PBS, the tissues or cells were homogenized in lysis buffer (50 mM Tris/HCl pH 6.8, 50 mM NaCl, 10% glycerol, 4 mM EDTA, 3 mM dithiothreitol, protease inhibitors, and phosphatase inhibitors I and II) on ice and then centrifuged twice to remove lipids and debris. The supernatant was used as the protein extract and the protein content was measured. Proteins (30 μg) were separated by SDS-PAGE and then transferred onto a nitrocellulose membrane (Bio Rad, Hercules, CA). The membrane was incubated with the antibodies (1:1,000 dilution) in 5% skim milk Tris buffered saline pH 7.4 at 4°C overnight and then visualized with a 1:10,000 dilution of horseradish peroxidase-linked IgG secondary antibody at room temperature for 2 h. The complexes were detected using the ECL chemiluminescence detection system, and expression ratios were calculated based on densitometric quantification of the bands, as described previously [34].

### RNA extraction and quantitative PCR

Total RNA of AT (approximately 100 mg), adipocytes (200 μL volume), or 3T3L-1 cells was isolated with 1 mL of TRI Reagent (Sigma-Aldrich) and centrifuged to remove lipids and insoluble substances according to the manufacturer’s protocol. After DNase I (Promega, Madison, WI) treatment to remove any contaminating genomic DNA from the RNA preparations, 1.5 μg of total RNA was reverse transcribed using random priming and Multiscribe reverse transcriptase according to the manufacturer’s instructions (Applied Biosystems).

For measurements of leptin, Mas receptor, MrgD receptor, and β-actin of rat AT and adipocytes, cDNA was diluted to 50, 25, and 12.5 ng per tube, and used for real time quantitative PCR analysis with Brilliant II QPCR Master Mix (Agilent Technologies) and Mx3000P (Agilent Technologies). The specific primers and Taqman probes were purchased from Applied Biosystems. The PCR conditions for the expression analysis were: 40 cycles of 95°C for 30 s, 55°C for 60 s and 72°C for 30 s. RT-PCR probes specific for rat leptin (Hs 01003372), rat β-actin (Hs 00164932), rat Mas (Rn 00562673), rat MrgD (Rn 0785783), rat iNOS (Rn 00561646), and rat plasminogen activator inhibitor type 1 (Rn 01481341) were purchased from Applied Biosystems.

For the measurements of leptin and β-actin in mouse 3T3L-1 cells, cDNA was diluted to 50, 25, and 12.5 ng per tube, and used for real time quantitative PCR analysis with Brilliant II SYBR green Master Mix (Agilent Technologies) and Mx3000P (Agilent Technologies). The PCR conditions for the expression analysis of mouse leptin and mouse β-actin were: 40 cycles of 94°C for 25 s, 60°C for 25 s and 72°C for 40 s. For the expression analysis of other proteins, the PCR conditions were: 45 cycles of 94°C for 25 s, 60°C for 40 s, and 72°C for 100 s. The specific primers used for PCR are as follows: for mouse leptin, 5’-TCCAGAAAGTCCAGGATGACAC-3’ (forward) and 5’-CACATTTTGGGAAGGCAGG-3’ (reverse) (GenBank accession no. NM_00849313); for mouse β-actin, 5’-AGCCATGTACGTAGCCATCC-3’ (forwards) and 5’-TCCCTCTCAGCTGTGGTGAA-3’ (reverse) (GenBank accession no. NM_007393).

### Nitric oxide assay

The measurement of nitric oxide was performed using a nitric oxide detection kit (Enzo Life Sciences Inc., Famingdale, NY) according to the manufacturer’s protocol. Medium exchange and addition of alamandine were performed at the same time. After 24 h incubation and subsequent washing of adipocytes (200 μL volume) with PBS, the adipocytes were immediately stored at -80°C. Within a few days, the adipocytes were homogenized in 500 μL of lysis buffer (50 mM Tris/HCl pH 6.8, 50 mM NaCl, 10% glycerol, 4 mM EDTA) on ice and then centrifuged twice to remove lipids and debris. The supernatant was used as the cell extract and the protein content was measured for normalization. The cell extract was diluted 8-fold using the detection kit’s reaction buffer. The extract was then filtered using a polysulfone filter (10,000 molecular weight cut off; Merck Millipore Ltd., Tullorgreen, Ireland). The filtered extract was promptly used as the sample in the detection kit.

### Data analysis

Western blotting was performed at least 3 times with single or duplicate samples. All other experiments were performed in duplicate or triplicate and repeated 3 times, unless otherwise stated. Data are presented as means ± SEM of at least three separate experiments. The significance of intergroup differences was determined by ANOVA. Values of *P*<0.05 were considered to indicate a significant difference.

## Results

### Ang1-7 alters leptin expression and secretion in adipose tissue (AT)

**Fig 1:**
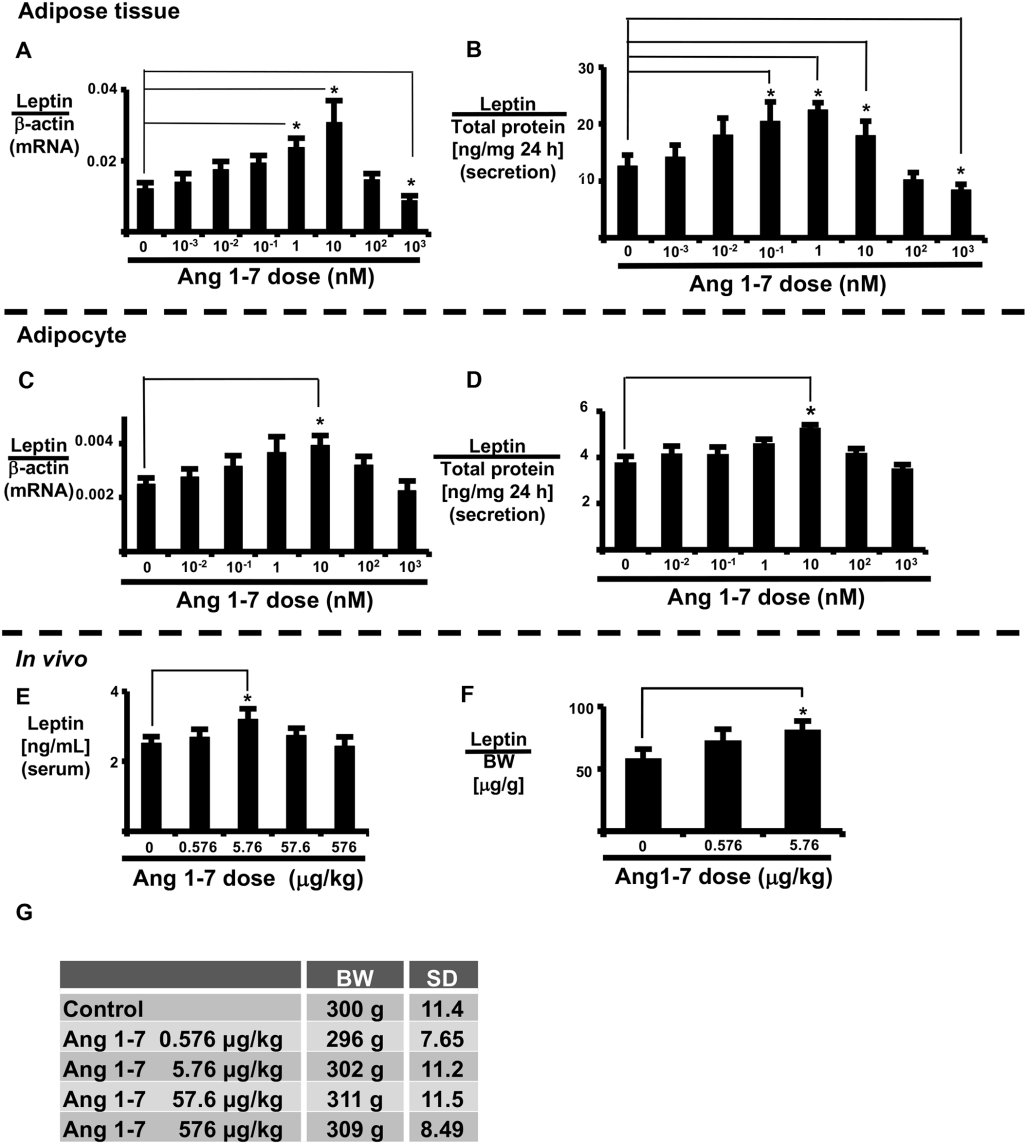
Effects of Ang1-7 *in vitro* and *in vivo*. Ang1-7 dose-response of leptin expression in AT (A, B) and isolated adipocytes (C, D). AT and isolated adipocytes were incubated with Ang1-7 for 24 h. Leptin mRNA expression (A, C) and secreted leptin (B, D) were measured as described in the Materials and Methods. Ang1-7 was administered intra-peritoneally over a 2-day period. Blood serum and peri-renal AT were collected 24 h later. Serum leptin levels (E) and leptin content in peri-renal AT (F) were measured as described in the Materials and Methods. Rat body weights (BW) at the time of blood sampling (G). Each column and bar represents the mean ± SEM of three separate experiments. An asterisk (*) indicates P<0.05 vs. vehicle. Secretion levels of leptin were normalized to total adipocyte protein, and expression of leptin mRNA was normalized to that of β-actin. AT leptin content was normalized to body weight.

We examined the effect of Ang1-7 on the expression and secretion of leptin in AT and isolated adipocytes (Fig 1A–1D). Ang1-7 increased leptin expression and secretion in a dose-dependent manner in both AT and isolated adipocytes, with maximal effects observed at 10 nM. Ang1-7 (10 nM) significantly increased leptin mRNA expression in AT (Fig 1A). Conversely, Ang1-7 reduced leptin expression and secretion in both AT and isolated adipocytes at concentrations ≥ 100 nM. Furthermore, 1000 nM Ang1-7 significantly reduced leptin mRNA expression and leptin secretion in AT (Fig 1A and 1B).

### Ang1-7 increases rat blood leptin levels

Next, we examined whether Ang1-7 increases blood leptin levels *in vivo* (Fig 1E and 1D). Systemic administration of Ang1-7 (intraperitoneal) did not alter body weight, but increased blood serum leptin levels (vehicle: 2.51 ± 0.195 ng/mL vs. Ang1-7 5.76 μg/kg: 3.20 ± 0.308 ng/mL) (Fig 1E) as well as leptin content in peri-renal AT (Fig 1F). From these results, low dose Ang1-7 (5.67 μg/kg) induced both secretion and expression of leptin *in vivo*.

### High levels of Ang1-7 suppress leptin expression via MrgD receptors in AT

**Fig 2:**
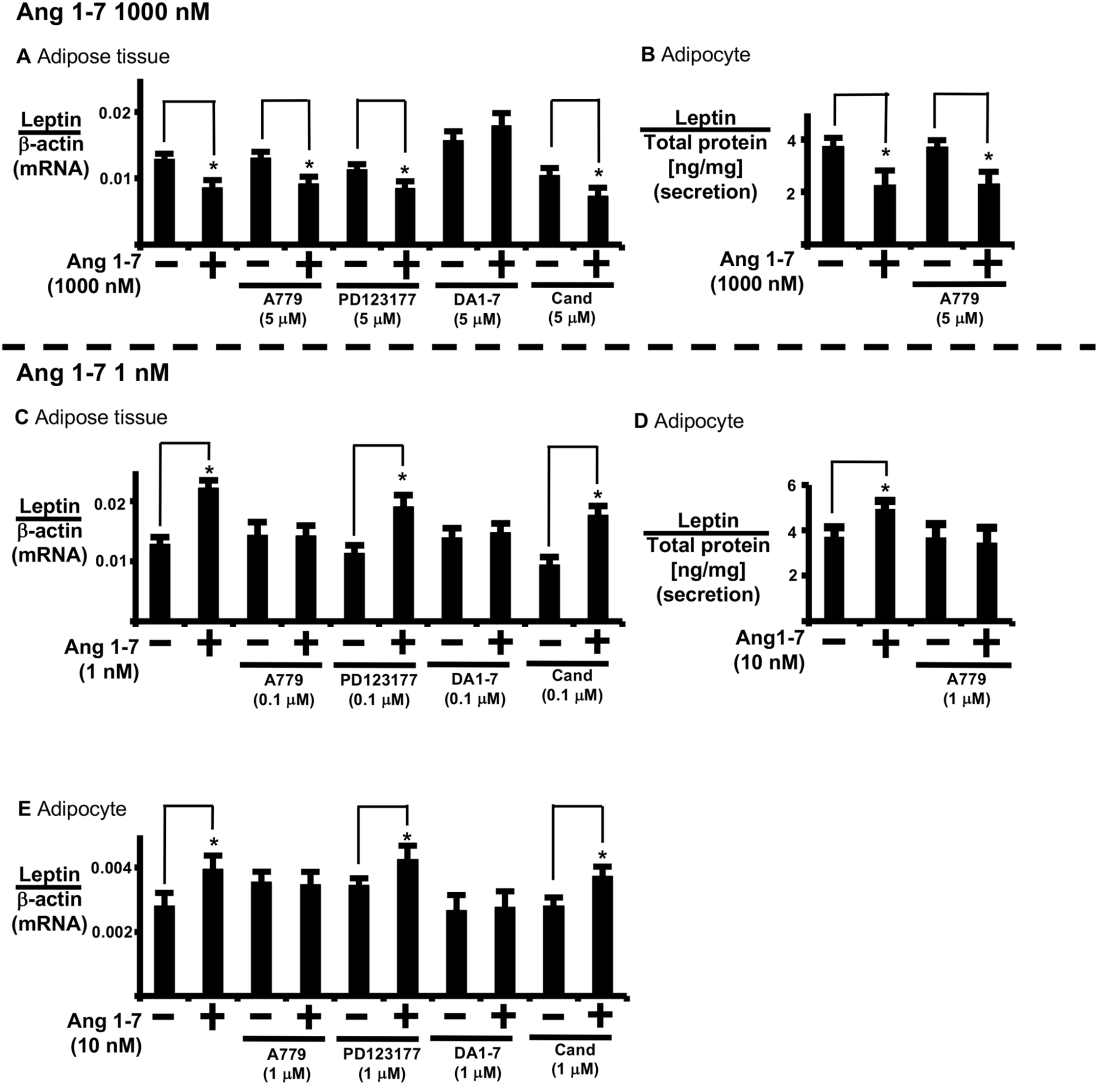
Analysis of receptor types involved in high- and low-dose Ang1-7 regulation of leptin expression. (A, C) AT and (E) isolated adipocytes were pre-treated with A779, PD123177, D-Pro^7^Ang1-7 (DA1-7), or candesartan (Cand) for 1 h prior to Ang1-7 addition. Control cells were treated with PBS for 24 h. The cells were incubated for 24 h prior to measuring leptin mRNA expression. (B, D) isolated adipocytes were pre-treated with A779 for 1 h prior to Ang1-7 addition, and incubated for 24 h prior to measuring leptin secretion. Secreted leptin was measured as described in the Materials and Methods of the supplemental data. Each column and bar represents the mean ± SEM of three separate experiments. An asterisk (*) indicates P<0.05 vs. vehicle. Leptin secretion levels were normalized to total adipocyte protein, and expression of leptin mRNA was normalized to that of β-actin.

We examined the contribution of the Mas receptor to leptin expression at high Ang1-7 concentrations (1000 nM) (Fig 2A and 2B). For this purpose, we assayed the effects of antagonist A779 (Mas receptor antagonist), PD123177 (AT2 receptor antagonist), candesartan (ATR1 receptor antagonist), and D-Pro^7^ Ang1-7 (Mas and MrgD receptor antagonist) (S1C–S1F Fig). D-Pro^7^ Ang1-7 reversed high-dose Ang1-7-induced regulation of leptin expression in AT. Conversely, A779, PD123177, and candesartan did not reverse high-dose Ang1-7-induced regulation of leptin secretion in AT (Fig 2A) and isolated adipocytes (Fig 2E).

In addition, A779 reversed low-dose Ang1-7 (1 nM)-induced regulation of leptin expression in AT (Fig 2C).

We used 3T3L-1 adipocytes overexpressing Mas or MrgD receptors to examine the contribution of the Mas receptor to leptin expression at high concentrations of Ang1-7. Ang1-7 induced leptin expression in a dose-dependent manner in Mas receptor-overexpressing 3T3L-1 adipocytes, up to 1000 nM Ang1-7 (high-dose). Also, A779 reversed leptin expression induced by low and high Ang1-7 doses in Mas receptor-overexpressing 3T3L-1 adipocytes (S2A Fig).

In comparison, 1000 nM Ang1-7 suppressed leptin expression in MrgD receptor-overexpressing 3T3L-1 adipocytes (S3A Fig). Also, alamandine did not have a significant influence on leptin expression in Mas receptor-overexpressing 3T3L-1 adipocytes (S2B Fig). Based on these results, high-dose (1000 nM) Ang1-7 suppressed leptin secretion and expression via MrgD receptors in AT.

### Alamandine suppresses expression and secretion of leptin in AT and isolated adipocytes

**Fig 3:**
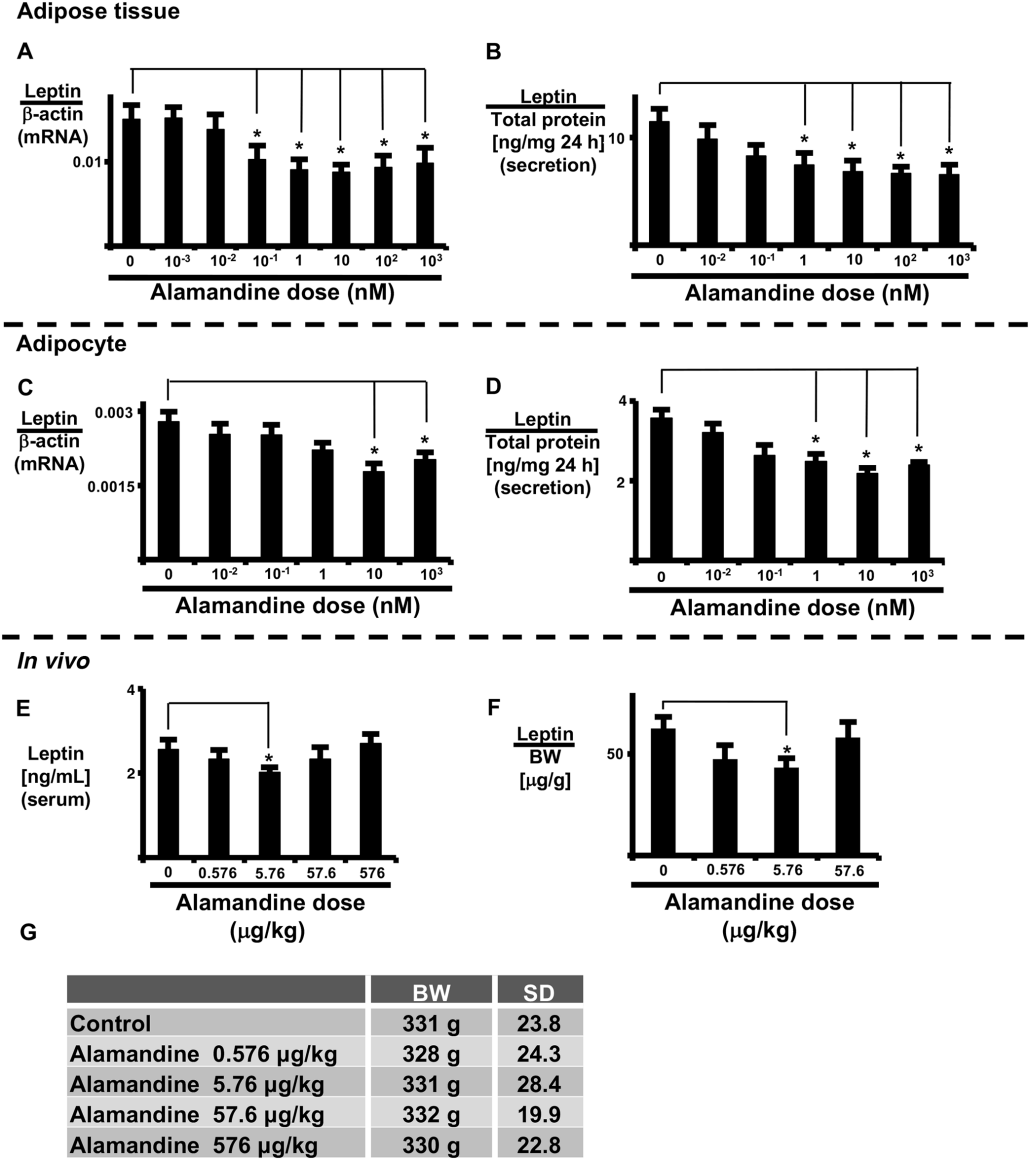
Effects of alamandine *in vitro* and *in vivo*. Alamandine dose-response of leptin expression in AT (A, B) and isolated adipocytes (C, D). AT and isolated adipocytes were incubated with alamandine for 24 h and leptin mRNA expression (A, C) and secreted leptin (B, D) were measured as described in the Materials and Methods. Alamandine was administered intra-peritoneally over a 2-day period. Serum leptin levels (E) and leptin content in peri-renal AT (F) were measured as described in the Materials and Methods. Rat body weights (BW) at the time of blood sampling (G). Each column and bar represents the mean ± SEM of three separate experiments. An asterisk (*) indicates P<0.05 vs. vehicle. Secretion levels of leptin were normalized to total adipocyte protein, and the expression of leptin mRNA was normalized to that of β-actin. Leptin content in AT was normalized to body weight.

Alamandine is the MrgD receptor ligand. Therefore, we examined whether alamandine reduces leptin expression and secretion in AT. AT or isolated adipocytes were exposed to alamandine for 24 h prior to sample collection. Alamandine significantly decreased leptin mRNA expression and leptin secretion in AT and isolated adipocytes (Fig 3A–3D) in a dose-dependent manner. Moreover, alamandine significantly decreased leptin mRNA expression in AT in a time-dependent manner (S4A Fig).

### Alamandine suppresses rat blood leptin levels

Next, we examined whether alamandine suppresses blood leptin levels *in vivo* (Fig 3E and 3F). Systemic administration (intraperitoneal) of alamandine did not alter body weight, but did reduce blood serum leptin levels (vehicle: 2.56 ± 0.240 ng/mL vs. alamandine 5.76 μg/kg: 2.01 ± 0.120 ng/mL) (Fig 3E) as well as leptin content in peri-renal AT (Fig 3F). These results indicate that low-dose alamandine (5.76 μg/kg) reduced both the secretion and expression of leptin *in vivo*.

### Alamandine suppression of leptin expression is mediated by MrgD and Gq

**Fig 4:**
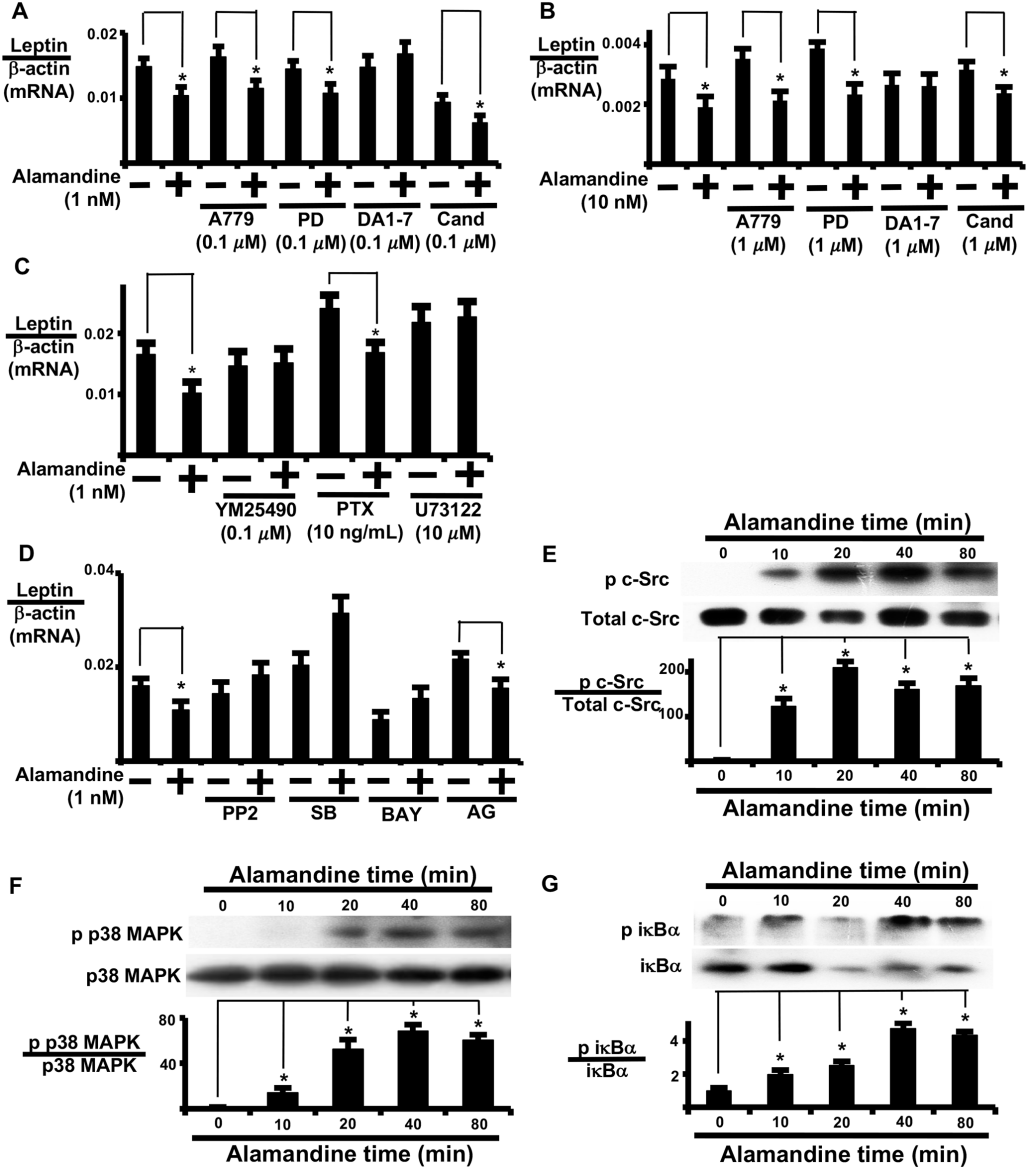
Analysis of receptor types and signal transduction involved in alamandine regulation of leptin expression. (A) AT and (B) isolated adipocytes were pre-treated with A779, PD123177 (PD), D-Pro^7^Ang1-7 (DA1-7), or candesartan (Cand) for 1 h prior to 24 h treatment with alamandine and subsequent analysis of leptin mRNA expression. Control cells were treated with PBS for 24 h. (C) AT was pre-treated with pertussis toxin (PTX) for 3 h, YM2590, or U73122 for 1 h prior to 24 h treatment with alamandine and subsequent analysis of leptin mRNA expression. (D) AT was pre-treated with PP2, SB239063 (SB), BAY11-7082 (BAY), or AG490 (AG) for 1 h prior to alamandine (1 nM) treatment and measurement of leptin mRNA expression 24 h later. Leptin mRNA levels were normalized to β-actin. (E) c-Src, (F) p38 MAP kinase, and (G) I*κ*Bα activation over time in AT. AT was incubated with alamandine (1 nM) for the indicated times to measure protein phosphorylation by western blotting. The ratio of phospho-protein to total protein was calculated based on densitometric quantification of the bands. Each column and bar represents the mean ± SEM of three separate experiments. An asterisk (*) indicates P<0.05 vs. vehicle tissue. The leptin mRNA levels were normalized to β-actin.

We examined whether MrgD, Mas, or ATR1 participates in the modulation of leptin expression by alamandine. D-Pro^7^ Ang1-7 reversed alamandine-induced regulation of leptin expression in AT (Fig 4A; S1C–S1F Fig) and isolated adipocytes (Fig 4B). These results suggest that alamandine regulates leptin expression through the MrgD receptor. Alamandine-induced leptin expression was reversed by a G_q/11_ protein inhibitor (YM25490), but not a G_i/o_ protein inhibitor (pertussis toxin) (Fig 4C). Furthermore, a phospholipase c inhibitor (U73122) was effective at inhibiting alamandine-induced leptin expression (Fig 4C), suggesting the involvement of phospholipase c in alamandine-induced leptin expression. YM25490 treatment also inhibited alamandine-induced c-Src activation (S5 Fig). Based on these results, alamandine induces changes in leptin expression by sequentially activating MrgD, G_q/11_, and phospholipase c.

### Involvement of c-Src, p38MAPK, and nuclear factor *κ*B

We used the following specific signal transduction inhibitors: PP2, SB239063, BAY11-7082, and AG490 (S1 Table). PP2, SB239063, or BAY11-7082 reversed alamandine-induced regulation of leptin expression (Fig 4D). Supporting the effects of signal transduction inhibitors, alamandine-induced phosphorylation of c-Src, p38 MAP kinase, and IκBα was assessed by western blotting (Fig 4E, 4F and 4G, respectively). We examined the order of MrgD-mediated signal transduction in AT with specific signal transduction inhibitors using phospho-reactive antibodies.

Bay11-7082 or SB23906 did not reverse alamandine-induced c-Src activation (S6A Fig). PP2 reversed alamandine-induced p38 MAP kinase activation (S6B Fig). Both Bay11-7082 and SB23096 reversed alamandine-induced I*κ*B*α* activation (S6C Fig). These results indicate that alamandine sequentially activated c-Src, p38 MAP kinase, and I*κ*B*α* in regulating leptin expression in AT.

### Alamandine induces inducible NOS (iNOS) and nitric oxide (NO) expression

**Fig 5:**
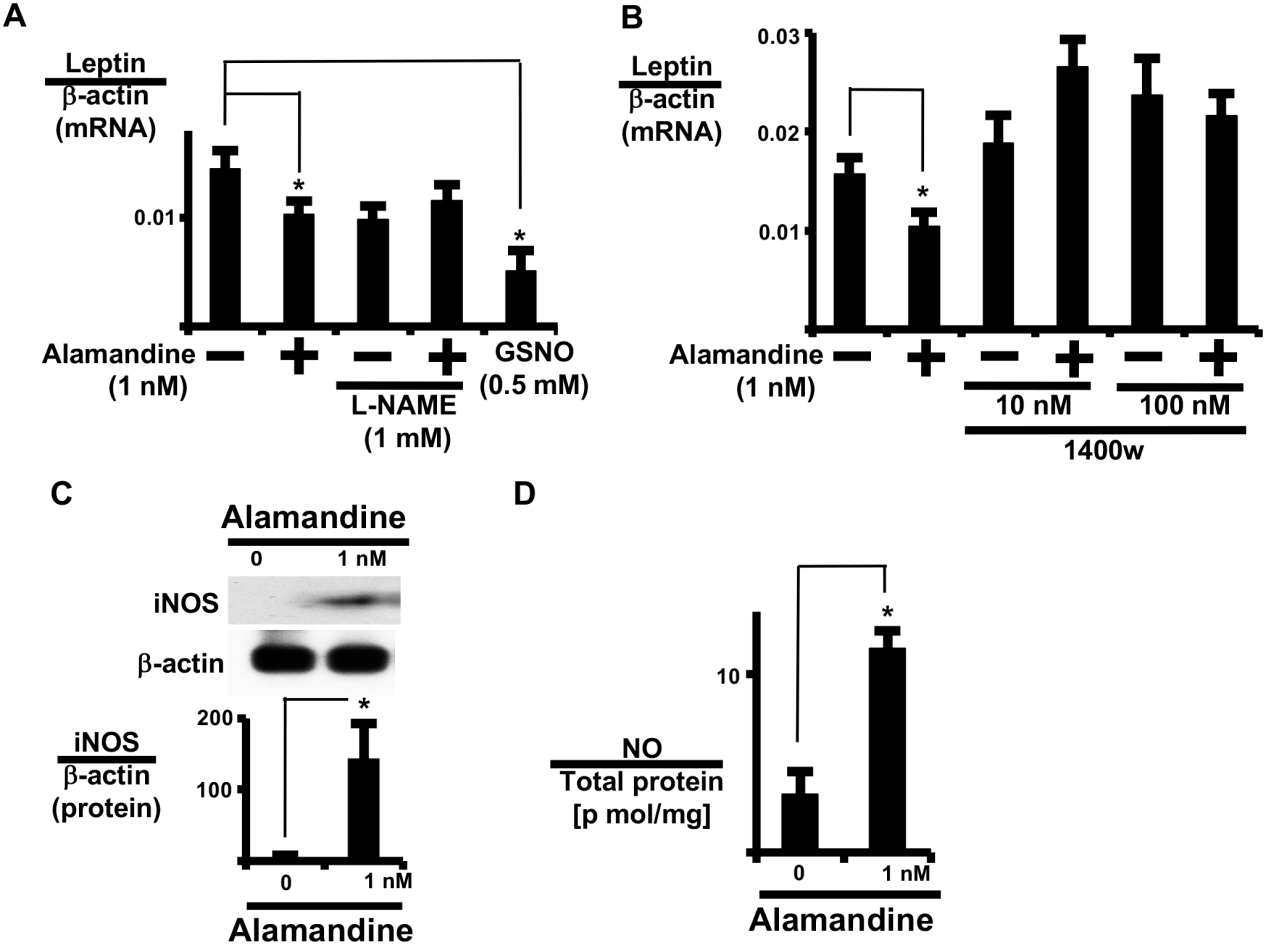
iNOS and NO mediate the effect of alamandine on leptin expression. (A) AT was pre-treated with N^G^-nitro-arginine methyl ester hydrochloride (L-NAME; 1 mM) for 1 h prior to alamandine or S-nitroso-L-glutathione (GSNO) and incubated for 24 h prior to measuring leptin mRNA expression. (B) AT was pre-treated with 1400w for 1 h prior to alamandine and incubated for 24 h prior to measuring leptin mRNA expression. (C) Isolated adipocytes were cultured with or without alamandine for 24 h prior to measuring iNOS expression by western blotting. The ratio of iNOS to β-actin was calculated based on densitometric quantification of the bands. (D) Isolated adipocytes were cultured with or without alamandine for 24 h prior to measuring NO. NO was measured as described in the Materials and Methods. Levels of NO were normalized to total adipocyte protein. Leptin and iNOS mRNA levels were normalized to β-actin. Each column and bar represents the mean ± SEM of three separate experiments. An asterisk (*) indicates P<0.05 vs. vehicle tissue.

Since cytotoxic signal transduction was activated by alamandine, we examined the involvement of iNOS expression and NO production (Fig 5A–5D). L-N^G^-nitroarginine methyl ester reversed the alamandine-induced reduction in leptin expression, and S-nitroso-L-glutathione, an NO donor, inhibited leptin expression (Fig 5A), suggesting the involvement of NOS signaling. Furthermore, an iNOS-selective inhibitor (1400w) reversed alamandine-induced leptin expression in AT (Fig 5B). Indeed, alamandine increased iNOS protein expression in adipocytes (Fig 5C). Thus, alamandine increased NO in isolated adipocytes (Fig 5D). Further, alamandine significantly increased iNOS mRNA expression in AT in a time-dependent manner, and the change in iNOS expression was earlier than the alamandine-induced change in leptin expression (S5A and S5C Fig). These results indicate that alamandine induced NO expression through iNOS expression in adipocytes.

### Alamandine induces inducible plasminogen activator inhibitor 1 (PAI-1) expression

**Fig 6:**
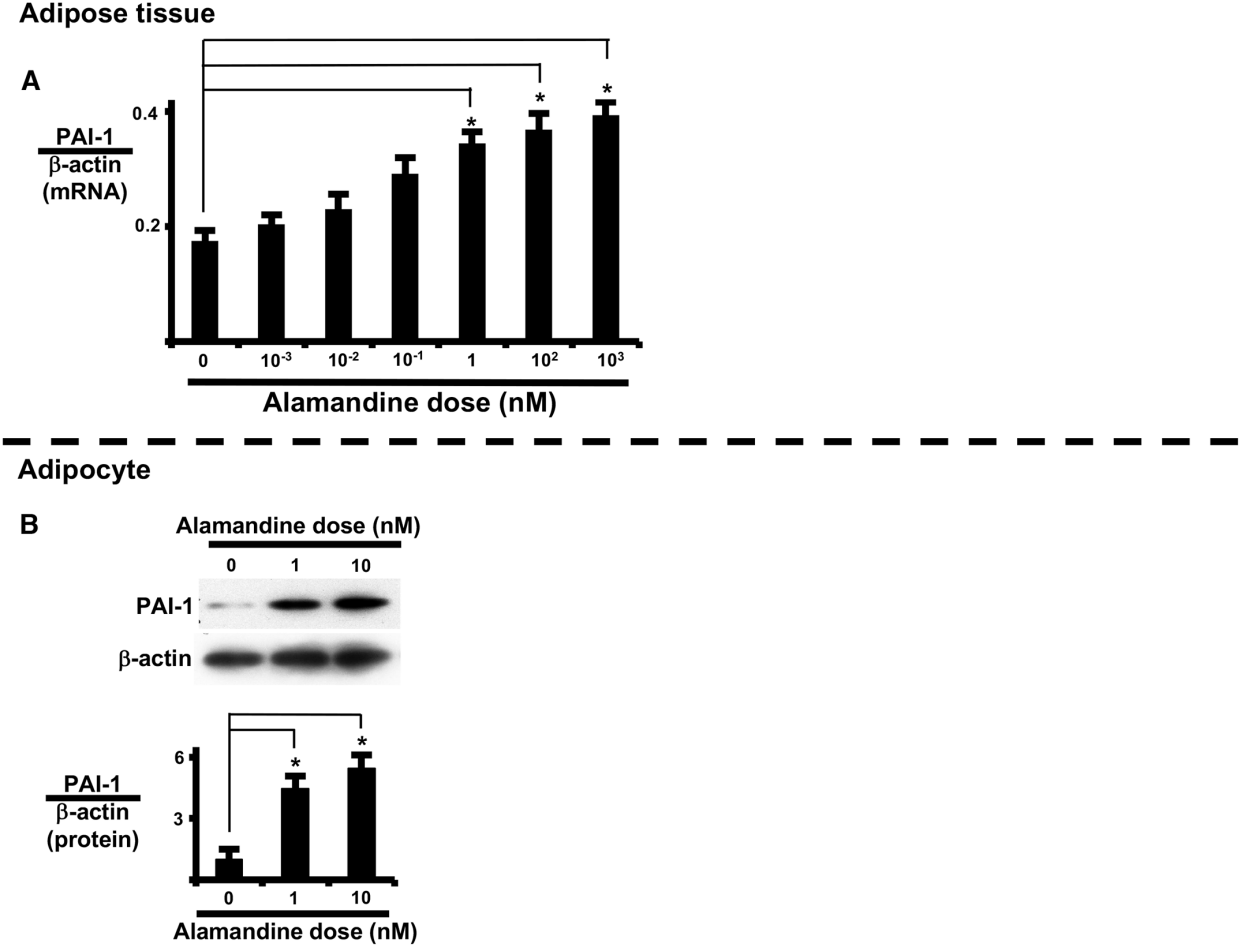
Alamandine induces PAI-1 expression. Alamandine dose-response of PAI-1 mRNA expression in AT (A) and of PAI-1 protein expression in isolated adipocytes (B). (A) AT was cultured with or without alamandine for 24 h prior to measuring PAI-1 mRNA expression. (B) Isolated adipocytes were cultured with or without alamandine for 24 h prior to measuring PAI-1 protein expression. PAI-1 mRNA and protein levels were normalized to β-actin. Each column and bar represents the mean ± SEM of three separate experiments. An asterisk (*) indicates P<0.05 vs. vehicle tissue.

Alamandine induced cytotoxic signal transduction and iNOS expression in AT and isolated adipocytes. Next, we examined the involvement of PAI-1 expression. Alamandine significantly increased PAI-1 mRNA expression in a dose-dependent manner in AT and PAI-1 protein expression in isolated adipocytes (Fig 6).

## Discussion

In this study, most of our experiments employed AT rather than isolated adipocytes. Adipocytes will enlarge if they are present within a skeleton of stromal fibrocytes. Therefore, when enlarged adipocytes are isolated using collagenase, the skeleton that is stabilizing the adipocyte disappears and the adipocytes rupture. For this reason, AT was adopted in the present study to investigate the function of adipocytes.

**Fig 7:**
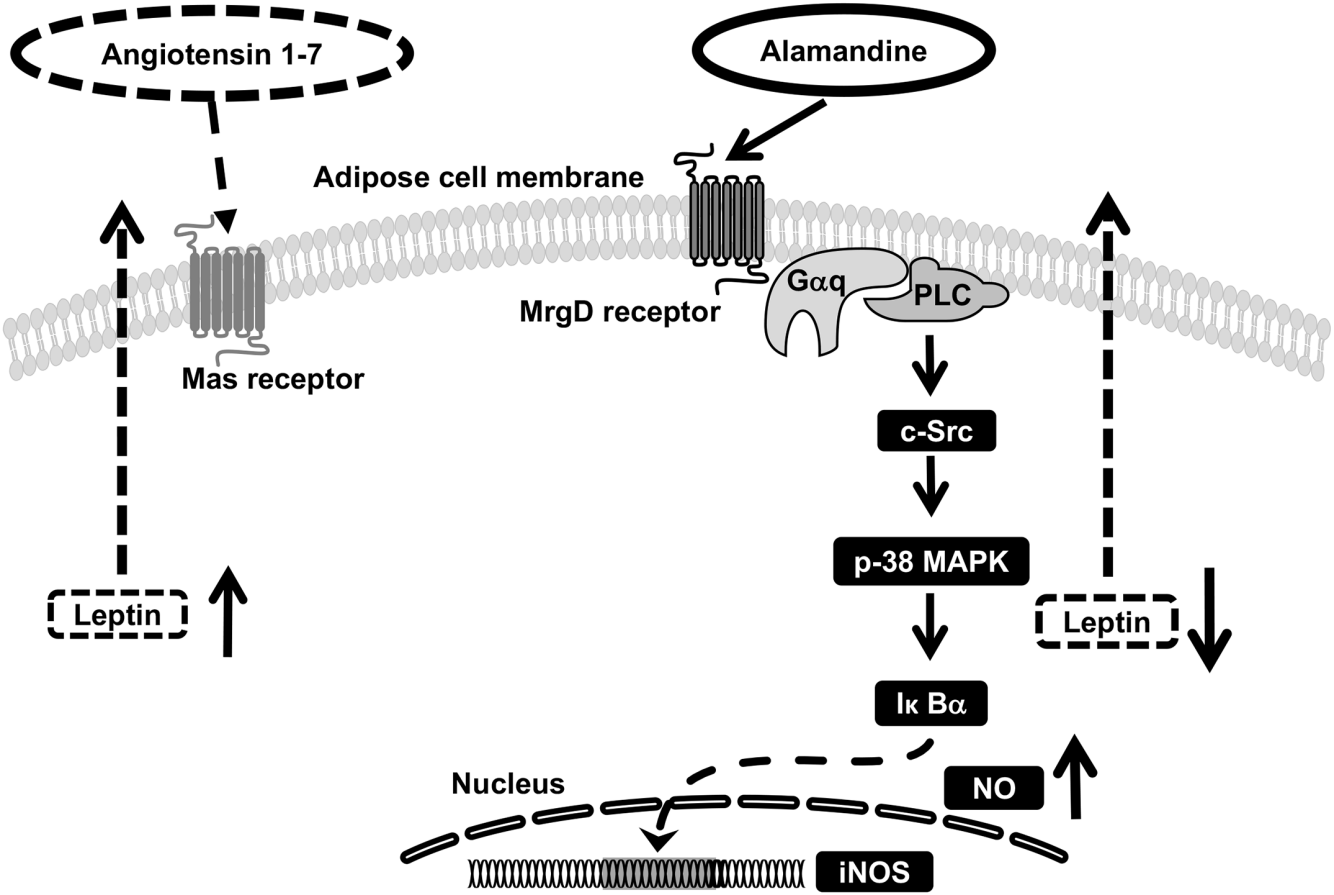
Postulated regulatory mechanism by alamandine of leptin expression in AT. Alamandine inhibits leptin expression, which is mediated by G_q/11_ and cytotoxic signal transduction pathways, ultimately resulting in the induction of NO expression. See text for more detail.

Alamandine and Ang1-7 are peptides of RAS. The Mas and MrgD receptors have opposing actions in adipose tissue, similar to AngII type 1 and type 2 receptors. That is, alamandine reduced the expression and secretion of leptin through cytotoxic signal transduction in peri-renal visceral AT (Fig 7) and enhanced the coagulation system by PAI-1 induction in peri-renal visceral AT.

AngII (precursor of Ang1-7) from 0.01 to 10 nM did not modify leptin expression in AT (S7 Fig). While high-dose alamandine did not reduce serum leptin levels *in vivo*, alamandine reduced leptin secretion and expression in AT and isolated adipocytes in a dose-dependent manner.

We suggest the following reasons for the observed *in vivo* and *in vitro* differences.

We examined whether high-dose alamandine binds to Mas receptors. High-dose alamandine did not induce leptin expression in Mas receptor-overexpressing 3T3TL-1 adipocytes (S2B Fig). Unlike angiotensin II, the metabolic cascade of alamandine is not well understood. While alamandine is known to be synthesized by angiotensin A, its fate is not well characterized. Thus, we propose that alamandine is modified, degraded or binds other receptors in adipocytes.

Ang1-7 induced leptin expression, while alamandine reduced leptin expression. Between Ang1-7 and alamandine, which has the predominant effect on leptin secretion in AT? Alamandine levels have not been determined in humans. AngII and angiotensin A are precursors of Ang1-7 and alamandine, respectively (S1B Fig). Both Ang1-7 and alamandine are synthesized by angiotensin enzyme 2. In people with normal renal converting function, the ratio of alamandine / Ang1-7 is expected to be about 0.2, which indicates that Ang1-7 predominates relative to alamandine under normal conditions [29]. Ang1-7 has a direct effect on vascular endothelial protection [14]. In renal failure patients, the ratio of alamandine / Ang1-7 is approximately 0.7. Further, in chronic kidney disease, blood levels of alamandine rise [29].

The MrgD receptor was originally discovered to be associated with nerve pain, and MrgD signal transduction remains unclear except for calcium release [10, 11] and phosphokinase A activation [37]. Moreover, the Mas and MrgD receptors similarly induce phosphokinase A and cAMP response element-binding phosphorylation in vascular smooth muscle cells, kidney mesangial cells, and human umbilical vein endothelial cells [37].

L-N^G^-nitroarginine methyl ester has been used to demonstrate that alamandine-induced vasodilation involves NO. However, the role of NOS has not been examined [5]. Ang1-7 stimulation of the Mas receptor suppresses Src activation in endothelial cells [12]. Conversely, we observed that alamandine stimulation of the MrgD receptor activates c-Src in AT. Furthermore, alamandine also induces iNOS expression in AT. NO synthesized by iNOS has a detrimental influence on adipocytes, such as mitochondrial dysfunction and lipoprotein lipase activity [38, 39]. Furthermore, alamandine activates NF*κ*B in AT, which results in increased expression of plasminogen activator inhibitor 1 in adipocytes [40]. In fact, we observed that alamandine induced PAI-1 expression in both AT and isolated adipocytes (Fig 6).

Alamandine showed opposing actions with respect to arteriosclerosis: alamandine suppressed sympathetic nerves through the reduction of leptin secretion but induced cytotoxic signal transduction in AT. In addition, meals and insulin also increase leptin secretion. Thus, the contribution of leptin suppression to arteriosclerosis may be low.

Similar to Ang1-7, alamandine is known to induce vasodilatation in the vascular endothelium [22]. We found that alamandine induced p38 MAP kinase activation and iNOS and PAI-1 expression in AT; however, alamandine reduced leptin secretion and expression in AT. These results suggest that alamandine may promote arteriosclerosis in AT despite its direct vasodilation effect.

Obesity is associated with an increased risk of diabetes mellitus, hypertension, and renal dysfunction. In the present study, one of the molecular mechanisms of arteriosclerosis in metabolic syndrome with renal dysfunction.

## Conclusions

Mas and MrgD receptors elicit opposing actions on leptin regulation in AT; specifically, leptin expression and secretion in AT were increased by Ang1-7 and decreased by alamandine. Alamandine activated cytotoxic signal transduction as well as iNOS and PAI-1 expression in AT.

## Supporting information

S1 Fig Amino acid sequences of the renin angiotensin system (RAS) components and schema of its receptor agonists. (A) Amino acid sequences and molecular weights of RAS components. (B) Schema of Mas and MrgD receptors in RAS. (C) Candesartan, AngII type 1 (AT1) receptor selective antagonist. (D) PD123177, AngII type 2 (AT2) receptor selective antagonist. (E) A779, Mas receptor selective antagonist. (F) D-Pro^7^Ang1-7, Mas and MrgD receptor antagonists.
(TIF)

S2 Fig Overexpression of Mas receptors in 3T3L-1 cells. (A) Effect of Ang1-7 on leptin expression in differentiated 3T3L-1 adipocytes overexpressing Mas receptors. 3T3L-1 adipocytes were pre-treated with A779 for 1 h prior to Ang1-7 addition. Control cells were treated with PBS for 24 h and incubated for 24 h prior to measuring leptin mRNA expression. (B) Effect of alamandine on leptin expression in differentiated 3T3L-1 adipocytes overexpressing Mas receptors. (C) Effect of Ang1-7 on leptin expression in differentiated 3T3L-1 cells overexpressing LacZ. (D) Expression of Mas receptor protein in Mas-overexpressing differentiated 3T3L-1 adipocytes. (E) Microscopic image of Mas receptor-overexpressing and LacZ-overexpressing differentiated 3T3L-1 adipocytes. Each column and bar represents the mean ± SEM of three separate experiments. An asterisk (*) indicates P<0.05 vs. vehicle. Expression of leptin mRNA was normalized to that of β-actin.
(TIF)

S3 Fig Overexpression of MrgD receptors in 3T3L-1 cells. (A) Effect of Ang1-7 on leptin expression in differentiated 3T3L-1 adipocytes overexpressing MrgD receptors. 3T3L-1 adipocytes were pre-treated with D-pro^7^ Ang1-7 (DA1-7; 5 μM) for 1 h prior to Ang1-7 (1000 nM) addition. Control cells were treated with PBS for 24 h and incubated for 24 h prior to measuring leptin mRNA expression. (B) Alamandine dose-response of leptin expression in MrgD receptor-overexpressing differentiated 3T3L-1 cells. 3T3L-1 adipocytes were pre-treated with D-pro^7^ Ang1-7 (DA1-7; 5 μM), or A779 (5 μM) for 1 h prior to alamandine (1000 nM) addition. Control cells were treated with PBS for 24 h and incubated for 24 h prior to measuring leptin mRNA expression. (C) Alamandine dose-response of leptin expression in LacZ-overexpressed differentiated 3T3L-1 cells. (D) Expression of MrgD receptor protein in MrgD-overexpressing differentiated 3T3L-1 adipocytes. (E) Microscopic image of MrgD receptor-overexpressing and LacZ-overexpressing differentiated 3T3L-1 adipocytes. Each column and bar represents the mean ± SEM of three separate experiments. An asterisk (*) indicates P<0.05 vs. vehicle. Expression of leptin mRNA was normalized to that of β-actin.
(TIF)

S4 Fig Time course of alamandine-induced changes in leptin, PAI-1, and iNOS expression in AT. AT was incubated with alamandine (1 nM) for 2, 4, 6, 12, 18, or 24 h to measure leptin mRNA expression. Each column and bar represents the mean ± SEM of three separate experiments. An asterisk (*) indicates p<0.05 vs. time 0. Expression of leptin mRNA was normalized to that of β-actin.
(TIF)

S5 Fig Analysis of G-proteins involved in c-Src activation during alamandine regulation of leptin expression. AT was pre-treated with pertussis toxin (PTX) (10 ng/mL) for 6 h or YM2590 (100 nM) for 1 h prior to alamandine addition, and incubated for 40 min prior to measuring c-Src phosphorylation by western blotting. Data are presented as the ratio of phospho-c-Src to non phospho-c-Src. Each column and bar represents the mean ± SEM of three separate experiments. An asterisk (*) indicates *P*<0.05 vs. vehicle tissue.
(TIF)

S6 Fig Analysis of the alamandine-stimulated MrgD receptor signal transduction sequence. AT was pre-treated with BAY11-7082 (BAY; 5 μM), SB239063 (SB; 2 μM), or PP2 (20 μM) for 1 h prior to alamandine (1 nM) addition, and incubated for 40 min prior to measuring protein phosphorylation by western blotting. (A) The ratio of phospho-c-Src to non-phospho c-Src, (B) the ratio of phospho-p38 MAP kinase to total p38 MAP kinase, and (C) the ratio of phospho-I*κ*Bα to total I*κ*Bα were calculated based on densitometric quantification of the bands. (D) Summary of results of signal transduction activation analysis. Each column and bar represents the mean ± SEM of three separate experiments. An asterisk (*) indicates *P*<0.05 vs. vehicle tissue.
(TIF)

S7 Fig Dose effect of AngII in AT. AT was incubated with AngII for 24 h. Each column and bar represents the mean ± SEM of three separate experiments. Expression of leptin mRNA was normalized to that of β-actin.
(TIF)

S1 Table List of reagents. Inhibitors, antagonists, and activator used in this study.
(TIF)
