# Supplementary figures and images for: Alamandine reduces leptin expression through the c-Src/p38 MAP kinase pathway in adipose tissue
Source: PLoS One. 2017 Jun 7;12(6):e0178769. doi: 10.1371/journal.pone.0178769 (PMC5462406; doi:10.1371/journal.pone.0178769)

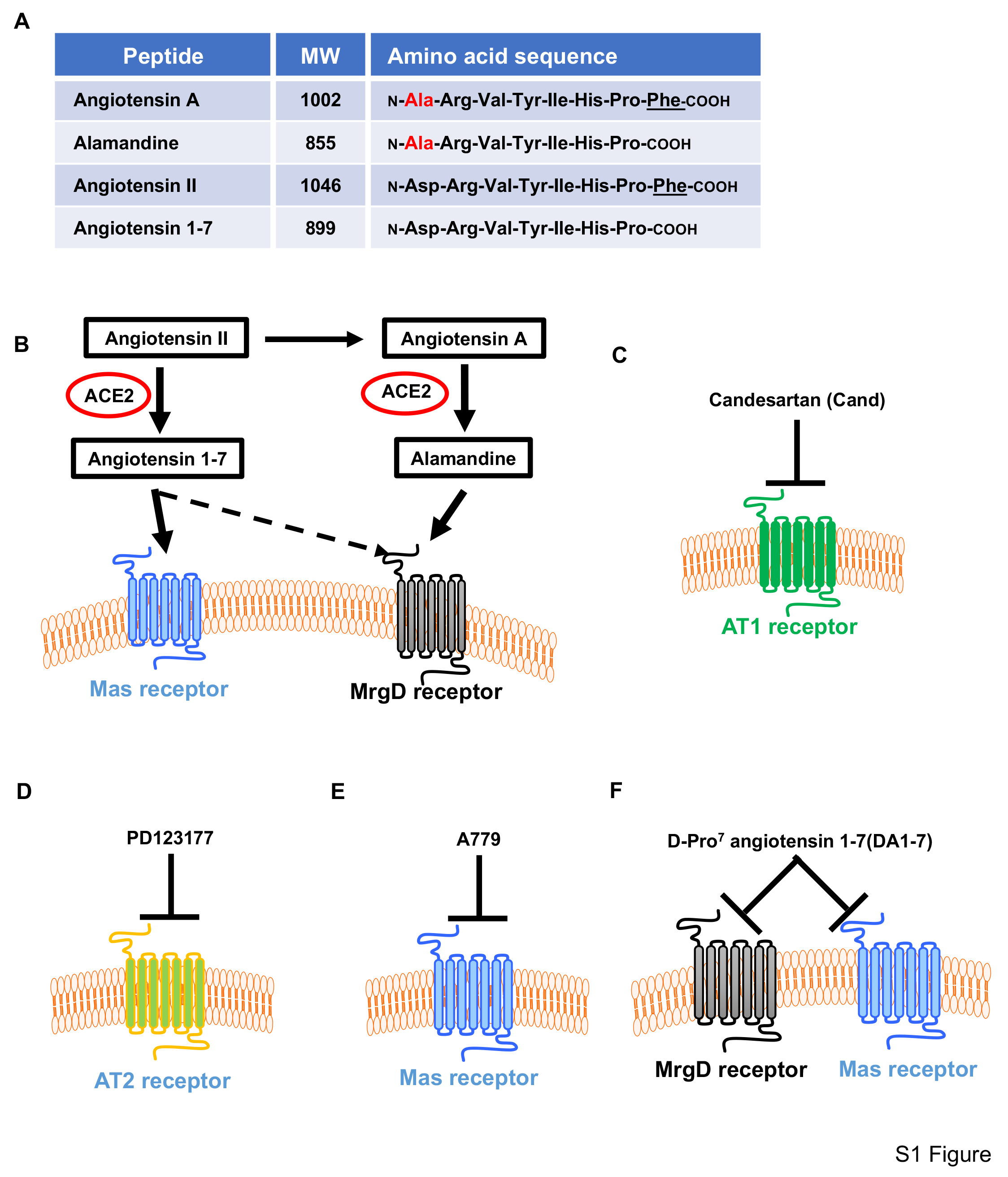

Supplement: S1 Fig — (A) Amino acid sequences and molecular weights of RAS components. (B) Schema of Mas and MrgD receptors in RAS. (C) Candesartan, AngII type 1 (AT1) receptor selective antagonist. (D) PD123177, AngII type 2 (AT2) receptor selective antagonist. (E) A779, Mas receptor selective antagonist. (F) D-Pro7Ang1-7, Mas and MrgD receptor antagonists. (TIF) [file pone.0178769.s001.tif]

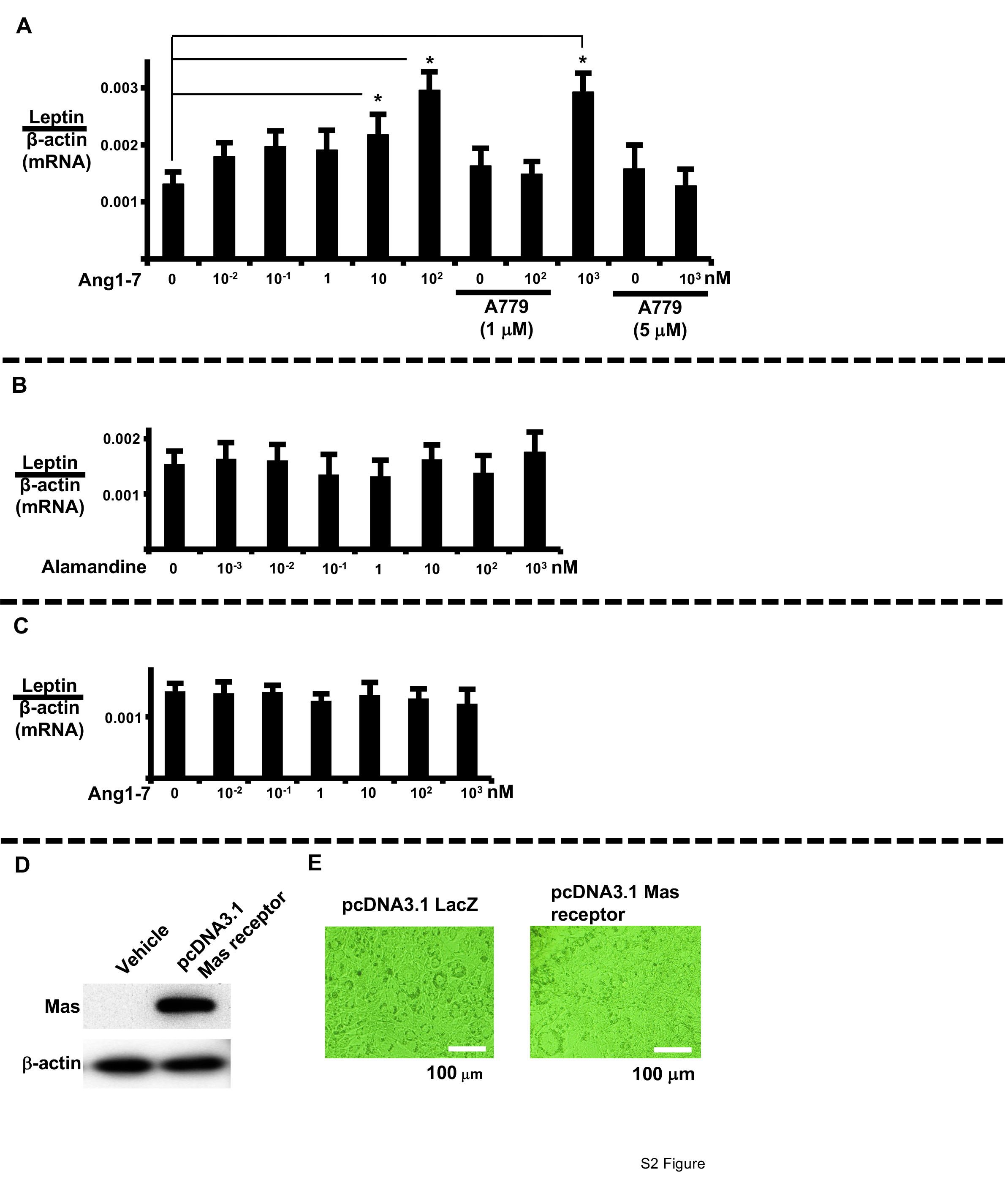

Supplement: S2 Fig — (A) Effect of Ang1-7 on leptin expression in differentiated 3T3L-1 adipocytes overexpressing Mas receptors. 3T3L-1 adipocytes were pre-treated with A779 for 1 h prior to Ang1-7 addition. Control cells were treated with PBS for 24 h and incubated for 24 h prior to measuring leptin mRNA expression. (B) Effect of alamandine on leptin expression in differentiated 3T3L-1 adipocytes overexpressing Mas receptors. (C) Effect of Ang1-7 on leptin expression in differentiated 3T3L-1 cells overexpressing LacZ. (D) Expression of Mas receptor protein in Mas-overexpressing differentiated 3T3L-1 adipocytes. (E) Microscopic image of Mas receptor-overexpressing and LacZ-overexpressing differentiated 3T3L-1 adipocytes. Each column and bar represents the mean ± SEM of three separate experiments. An asterisk (*) indicates P<0.05 vs. vehicle. Expression of leptin mRNA was normalized to that of β-actin. (TIF) [file pone.0178769.s002.tif]

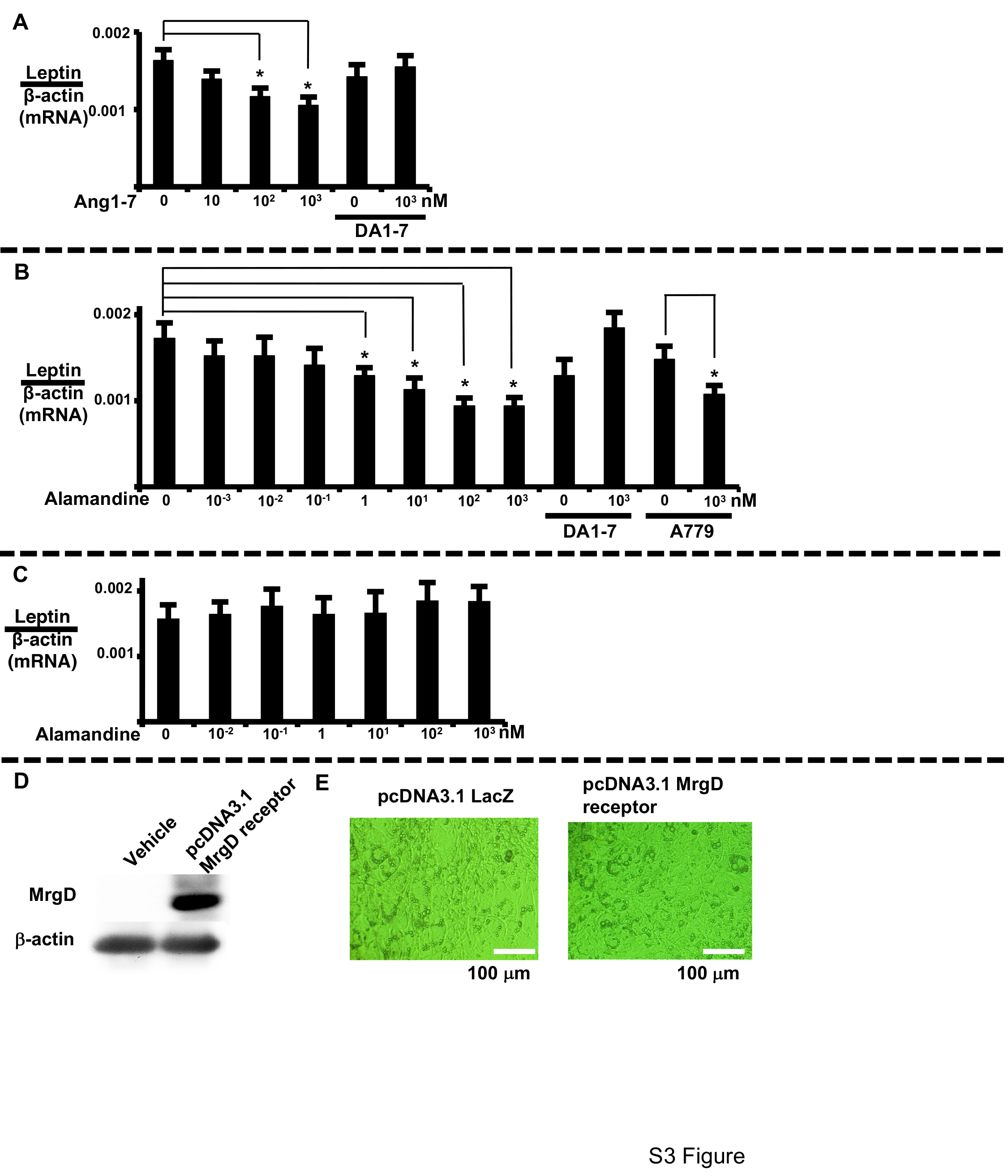

Supplement: S3 Fig — (A) Effect of Ang1-7 on leptin expression in differentiated 3T3L-1 adipocytes overexpressing MrgD receptors. 3T3L-1 adipocytes were pre-treated with D-pro7 Ang1-7 (DA1-7; 5 μM) for 1 h prior to Ang1-7 (1000 nM) addition. Control cells were treated with PBS for 24 h and incubated for 24 h prior to measuring leptin mRNA expression. (B) Alamandine dose-response of leptin expression in MrgD receptor-overexpressing differentiated 3T3L-1 cells. 3T3L-1 adipocytes were pre-treated with D-pro7 Ang1-7 (DA1-7; 5 μM), or A779 (5 μM) for 1 h prior to alamandine (1000 nM) addition. Control cells were treated with PBS for 24 h and incubated for 24 h prior to measuring leptin mRNA expression. (C) Alamandine dose-response of leptin expression in LacZ-overexpressed differentiated 3T3L-1 cells. (D) Expression of MrgD receptor protein in MrgD-overexpressing differentiated 3T3L-1 adipocytes. (E) Microscopic image of MrgD receptor-overexpressing and LacZ-overexpressing differentiated 3T3L-1 adipocytes. Each column and bar represents the mean ± SEM of three separate experiments. An asterisk (*) indicates P<0.05 vs. vehicle. Expression of leptin mRNA was normalized to that of β-actin. (TIF) [file pone.0178769.s003.tif]

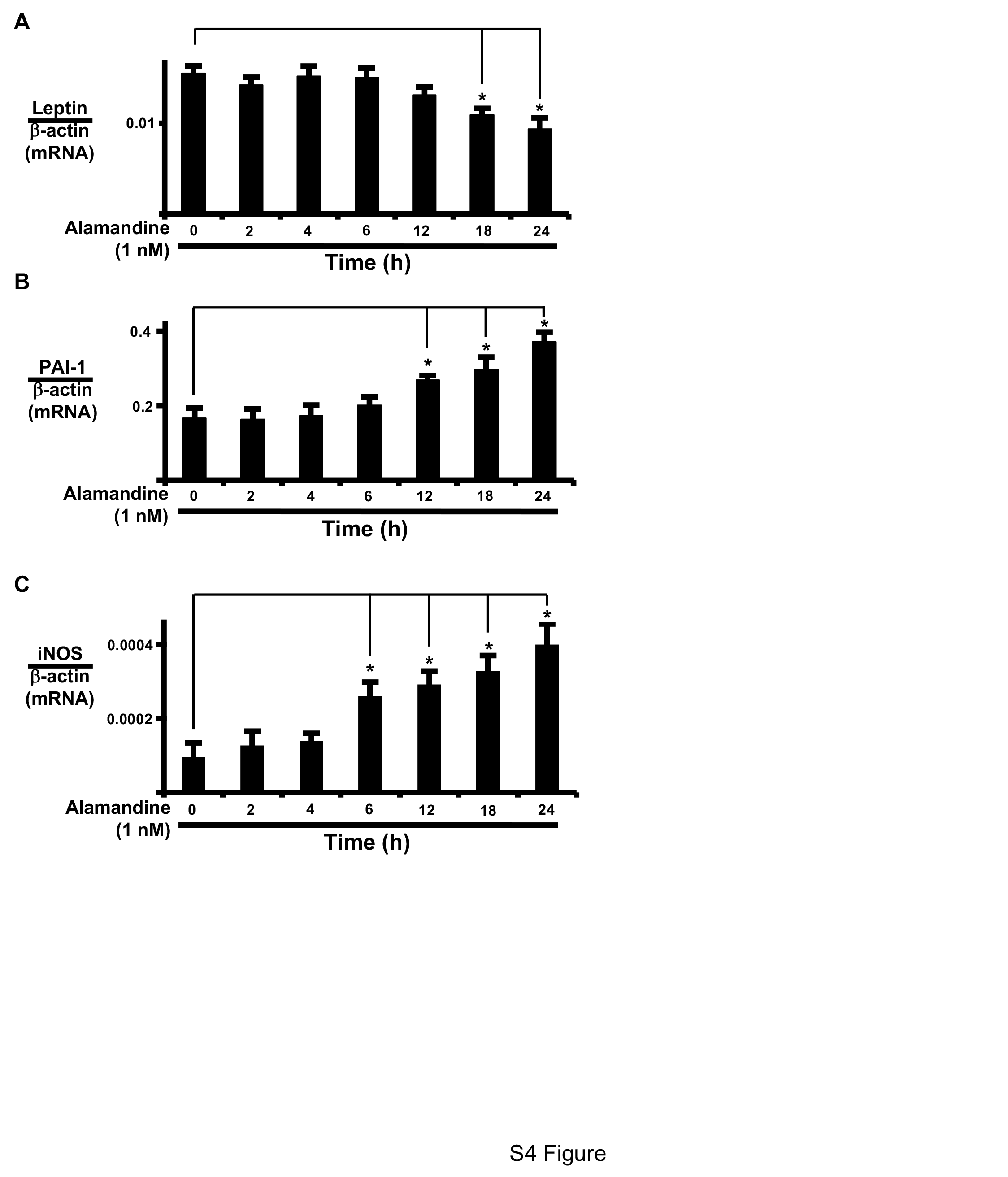

Supplement: S4 Fig — AT was incubated with alamandine (1 nM) for 2, 4, 6, 12, 18, or 24 h to measure leptin mRNA expression. Each column and bar represents the mean ± SEM of three separate experiments. An asterisk (*) indicates p<0.05 vs. time 0. Expression of leptin mRNA was normalized to that of β-actin. (TIF) [file pone.0178769.s004.tif]

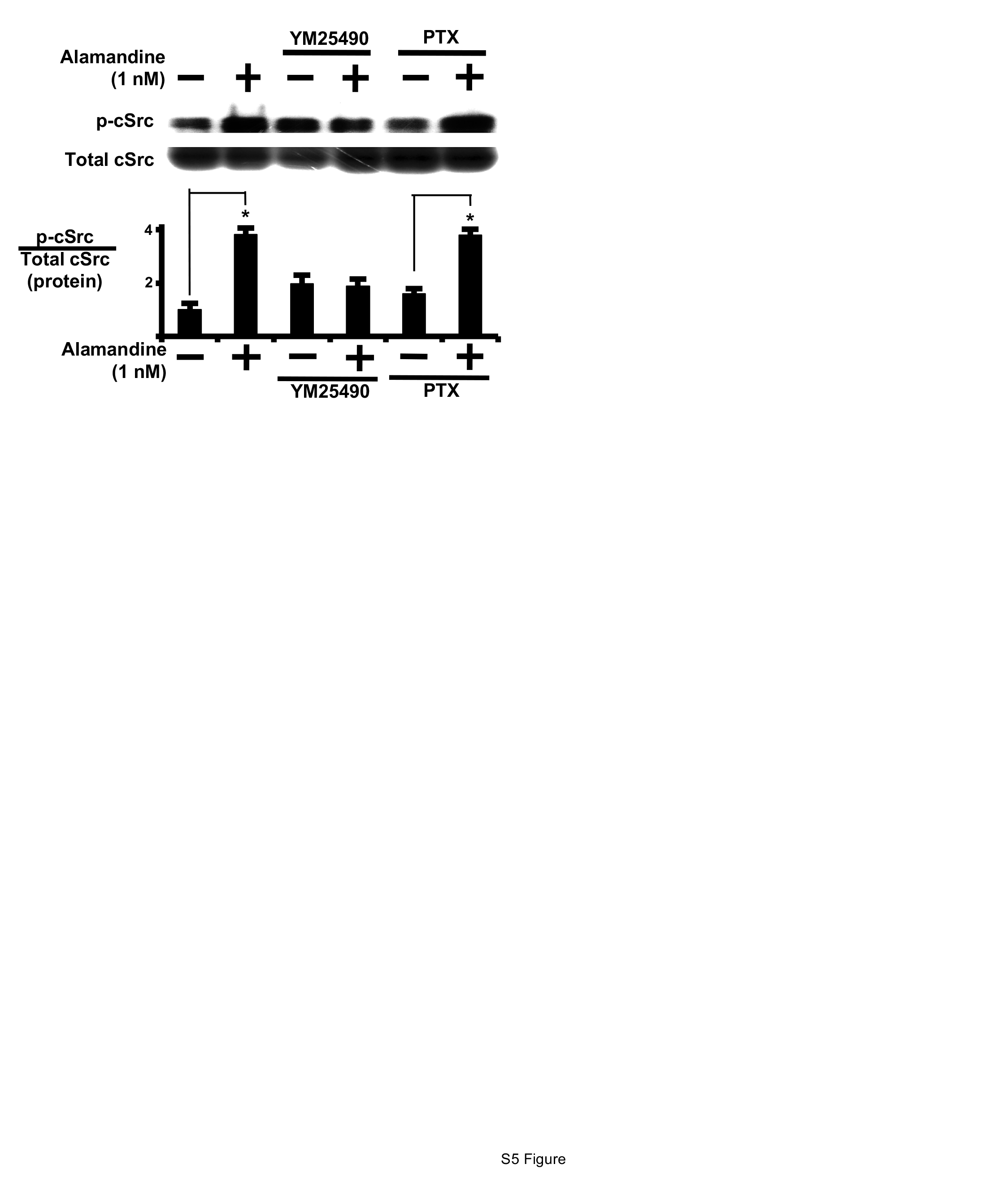

Supplement: S5 Fig — AT was pre-treated with pertussis toxin (PTX) (10 ng/mL) for 6 h or YM2590 (100 nM) for 1 h prior to alamandine addition, and incubated for 40 min prior to measuring c-Src phosphorylation by western blotting. Data are presented as the ratio of phospho-c-Src to non phospho-c-Src. Each column and bar represents the mean ± SEM of three separate experiments. An asterisk (*) indicates P<0.05 vs. vehicle tissue. (TIF) [file pone.0178769.s005.tif]

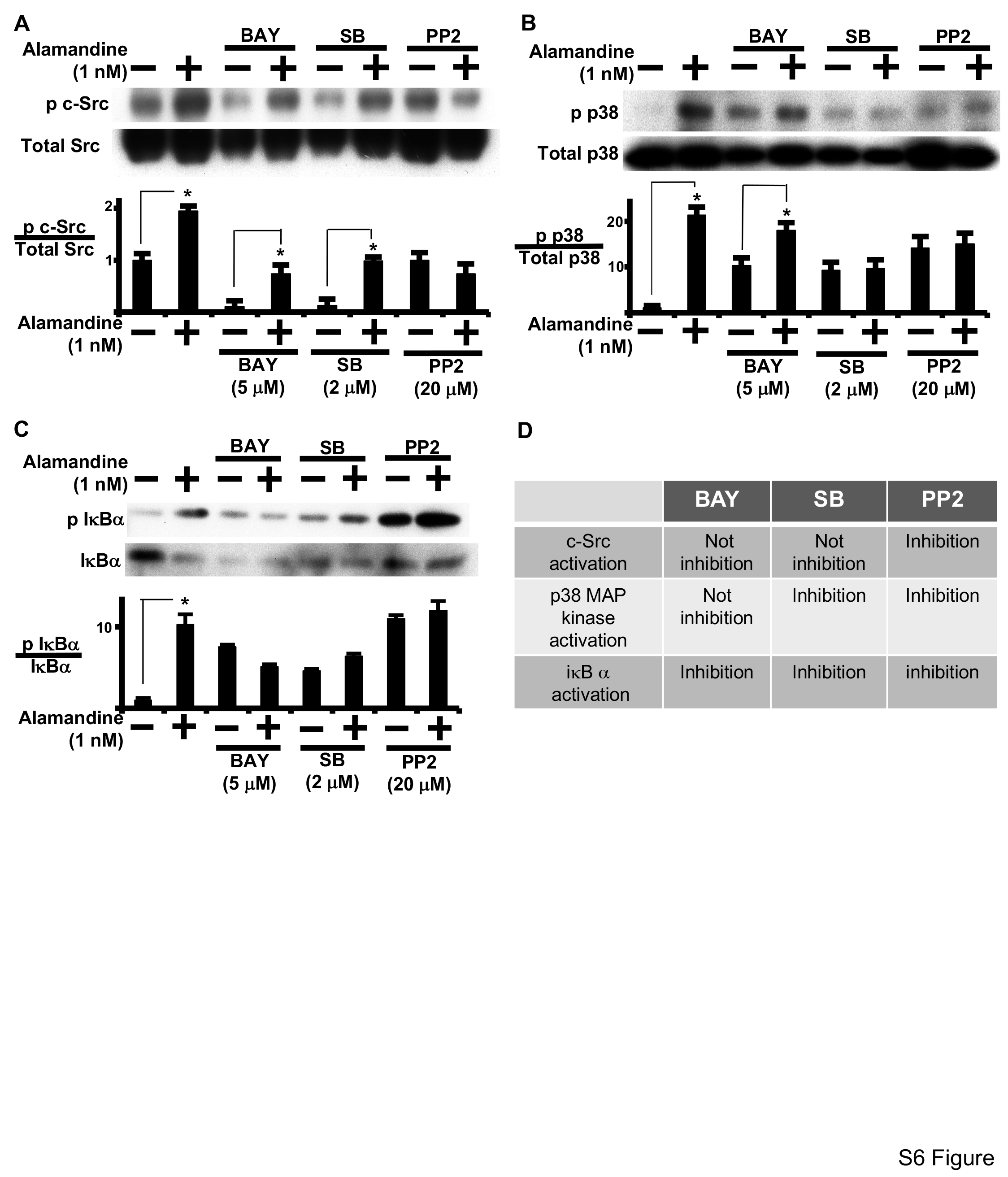

Supplement: S6 Fig — AT was pre-treated with BAY11-7082 (BAY; 5 μM), SB239063 (SB; 2 μM), or PP2 (20 μM) for 1 h prior to alamandine (1 nM) addition, and incubated for 40 min prior to measuring protein phosphorylation by western blotting. (A) The ratio of phospho-c-Src to non-phospho c-Src, (B) the ratio of phospho-p38 MAP kinase to total p38 MAP kinase, and (C) the ratio of phospho-IκBα to total IκBα were calculated based on densitometric quantification of the bands. (D) Summary of results of signal transduction activation analysis. Each column and bar represents the mean ± SEM of three separate experiments. An asterisk (*) indicates P<0.05 vs. vehicle tissue. (TIF) [file pone.0178769.s006.tif]

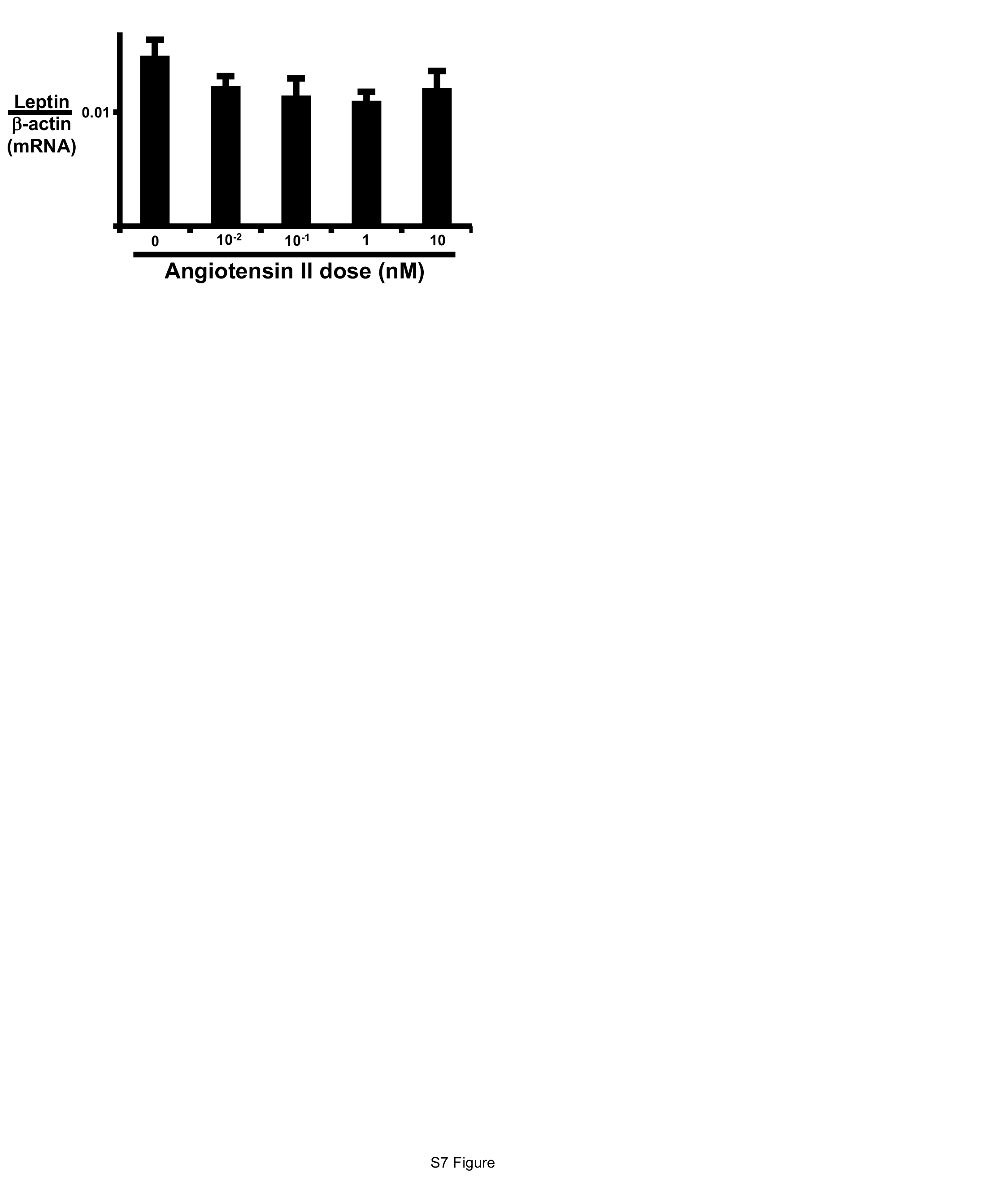

Supplement: S7 Fig — AT was incubated with AngII for 24 h. Each column and bar represents the mean ± SEM of three separate experiments. Expression of leptin mRNA was normalized to that of β-actin. (TIF) [file pone.0178769.s007.tif]

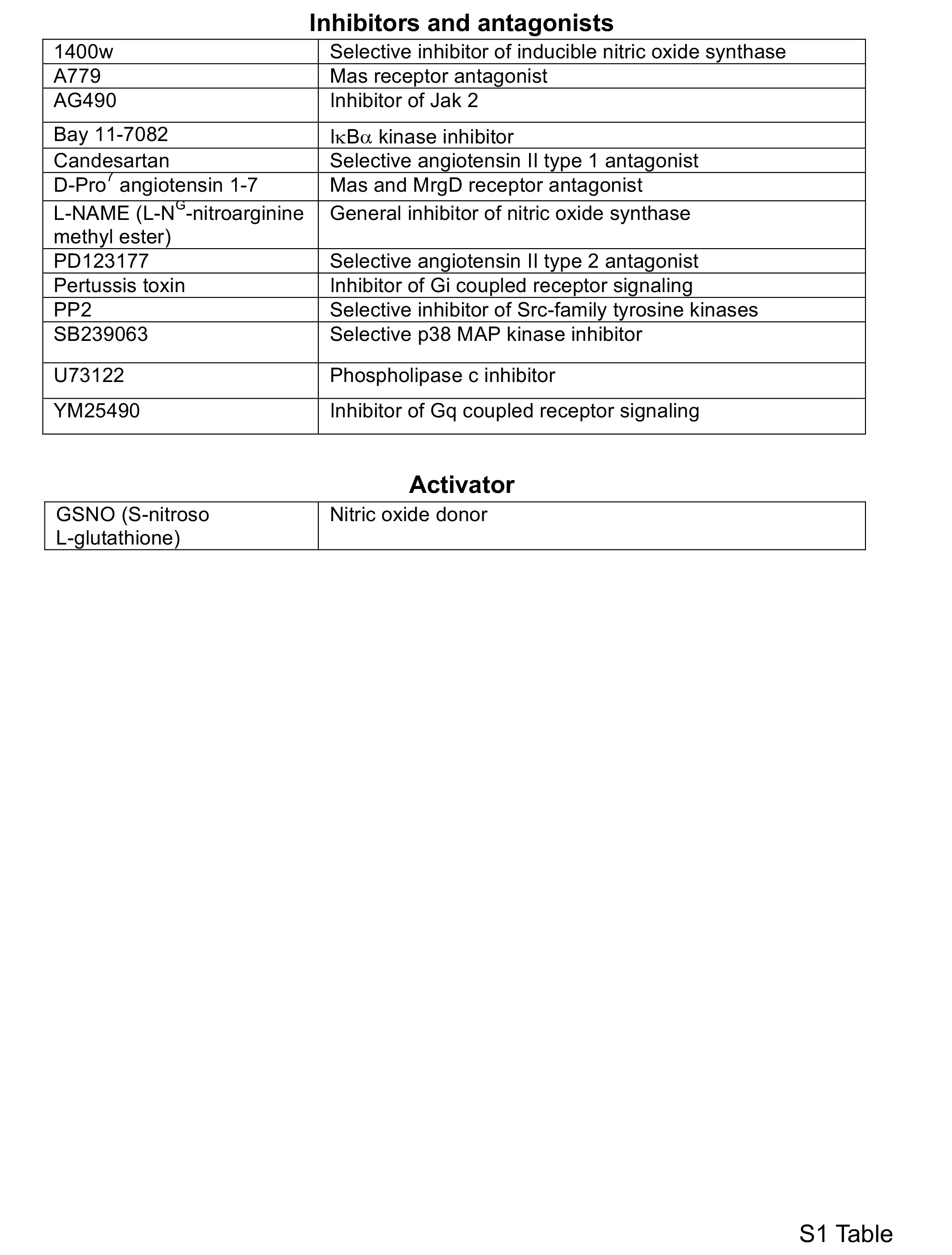

Supplement: S1 Table — Inhibitors, antagonists, and activator used in this study. (TIF) [file pone.0178769.s008.tif]
